# Effects of aerobic exercise on ectopic lipids in patients with growth hormone deficiency before and after growth hormone replacement therapy

**DOI:** 10.1038/srep19310

**Published:** 2016-01-21

**Authors:** Emanuel R. Christ, Andrea Egger, Sabin Allemann, Tania Buehler, Roland Kreis, Chris Boesch

**Affiliations:** 1 Division of Endocrinology, Diabetology and Clinical Nutrition, University Hospital of Bern, Inselspital, CH-3010 Bern, Switzerland; 2 Department of Clinical Research, Division of MR-Spectroscopy and Methodology, University of Bern, Inselspital, CH-3010 Bern, Switzerland; 3 Department of Internal Medicine, University Hospital of Basel, CH-4056 Basel, Switzerland

## Abstract

Growth hormone replacement therapy (GHRT) increases exercise capacity and insulin resistance while it decreases fat mass in growth hormone-deficient patients (GHD). Ectopic lipids (intramyocellular (IMCL) and intrahepatocellular lipids (IHCL) are related to insulin resistance. The effect of GHRT on ectopic lipids is unknown. It is hypothesized that exercise-induced utilization of ectopic lipids is significantly decreased in GHD patients and normalized by GHRT. GHD (4 females, 6 males) and age/gender/waist-matched control subjects (CS) were studied. VO_2max_ was assessed on a treadmill and insulin sensitivity determined by a two-step hyperinsulinaemic-euglycaemic clamp. Visceral (VAT) and subcutaneous (SAT) fat were quantified by MR-imaging. IHCL and IMCL were measured before and after a 2 h exercise at 50–60% of VO_2max_ using MR-spectroscopy (∆IMCL, ∆IHCL). Identical investigations were performed after 6 months of GHRT. VO_2max_ was similar in GHD and CS and significantly increased after GHRT; GHRT significantly decreased SAT and VAT. 2 h-exercise resulted in a decrease in IMCL (significant in CS and GHRT) and a significant increase in IHCL in CS and GHD pre and post GHRT. GHRT didn’t significantly impact on ∆IMCL and ∆IHCL. We conclude that aerobic exercise affects ectopic lipids in patients and controls. GHRT increases exercise capacity without influencing ectopic lipids.

Intramyocellular (IMCL) and intrahepatocellular (IHCL) lipids are so-called ectopic lipid depots. IMCL and IHCL and in particular their degradation products have been related to impaired insulin action in the respective tissues^1^,^2^. An acute aerobic 2 h physical exercise at 50% VO_2max_ has been shown to decrease IMCL and intramyocardiocellular lipids (ICCL) and increase IHCL in healthy physically active subjects^3^,^4^. These findings suggest that during physical exercise ectopic lipids are utilized in “working” tissue (skeletal muscle, heart muscle) whereas the increase in lipolysis - as evidenced by an increase in systemic free fatty acid concentrations (FFA) - results in a possible transitory storage of fat in the liver^5^,^6^. Importantly, IMCL appear to be particularly flexible energy stores in physically active healthy subjects^7^,^8^ whereas sedentary (overweight) subjects tend to have similarly high IMCL (like athletes, therefore called the athletic paradox), but are less capable to deplete these stores during physical exercise^8^. Based on these observations it is hypothesized that exercise-induced changes in ectopic fat depots are related to a) insulin sensitivity, b) fat mass (i.e. visceral and subcutaneous adipose tissue, VAT and SAT) and c) exercise capacity.

Hypopituitary patients with growth hormone deficiency (GHD) are characterized by a decreased exercise capacity and an increase in fat mass, in particular VAT^9^. A panoply of methods have been used to assess insulin resistance in these patients and the results suggest that these patients are modestly insulin resistant^9^. Growth hormone replacement therapy (GHRT) has been shown to improve exercise capacity^10^, decrease fat mass through its lipolytic action^9^ and further increase insulin resistance – at least at short time growth hormone (GH) exposure^9^. Thus, GHRT influences three potential parameters (i.e. exercise capacity, fat mass, insulin sensitivity) that may influence the flexibility of ectopic lipids. While GHRT is a therapy option for GHD patients, it can also serve as a model of insulin resistance and subsequent effects with general significance.

Importantly, previous studies that investigated metabolic characteristics in GHD patients before and after GHRT included control subjects (CS) who were matched for age, gender and BMI^9^ while waist circumference was usually not included in the matching criteria. However, waist circumference (correlated with VAT) in hypopituitary patients may be significantly influenced not only by GHD and GHRT, but – amongst others - also by the dose of hydrocortisone and potentially sex steroid replacement therapy^11^. Since a significant number of patients with GHD suffers also from secondary adrenal and gonadal insufficiency, it is conceivable that the insulin resistant condition, associated with an increase in VAT of patients with GHD, is rather due to an unphysiological pituitary hormone replacement therapy than to GHD *per se*. By matching waist circumference, an important confounding factor that influences insulin sensitivity^12^ and potentially ectopic lipids, is avoided.

The current study aimed at studying the flexibility of ectopic fat stores in patients with GHD before and after GHRT, and in sedentary healthy subjects matched for age, gender, BMI, and waist circumference. We hypothesized that, due to the lipolytic action of GH, GHRT results in an increase in the flexibility of ectopic lipids, thereby facilitating the energy supply and improving peak aerobic capacity. Ectopic lipids were non-invasively assessed using^1^H-MR-spectroscopy before and after an acute 2-h bout of exercise at 50% VO_2max_. Importantly, the potential regulatory factors of ectopic lipids, namely peak aerobic exercise capacity, fat mass (SAT and VAT) and insulin sensitivity (peripheral and hepatic) were investigated in patients before and after GHRT and in matched CS.

## Material and Methods

This prospective single-center study was performed at the University Hospital of Bern, Switzerland. All investigations were carried out at the Department of Clinical Research (Division of Endocrinology, Diabetes and Clinical Nutrition, and Division of MR-Spectroscopy and Methodology). The study was approved by the local review board (Kantonale Ethikkommission, Bern) and all subjects gave written informed consent. The study was performed according to the declaration of Helsinki, the guidelines of good clinical practice, and the Swiss health laws on clinical research (Clinical.Trials.gov: NCT00491582).

### Study participants

Four female and 6 male patients with severe GHD were recruited from the outpatient clinic. GHD was defined as a peak GH of less than 3 mU/l during an insulin tolerance test with nadir plasma glucose of less than 2.2 mmol/l and hypoglycemic symptoms^13^. Patients were included provided they had been under stable conventional hormone replacement therapy (glucocorticoids, thyroxin and sex hormones) as needed for at least 6 months and capable to exercise on a treadmill for 2 hours. Exclusion criteria were (former or present) ACTH- or GH-secreting pituitary adenoma, abnormal liver or renal function, active neoplasia, severe cardiovascular disease (unstable coronary artery disease, heart failure New York Heart Association III-IV), diabetes mellitus, hemophilia, therapy with drugs known to affect lipid or glucose metabolism, inability to exercise and contraindications to exposure to a 3 Tesla magnetic field. Additionally, ten sedentary subjects, each matched to one of the patients regarding age, gender, BMI and waist circumference were recruited.

#### Study Protocol

GHD patients and CS attended the hospital after an overnight fast. With regard to time point of the investigations, male patients with LH/FSH insufficiency were studied 6 weeks after the last i.m. testosterone administration whereas the female patients with LH/FSH-insufficiency were investigated during the administration of hormone replacement therapy. Female patients who were LH/FSH-sufficient were investigated during the follicular phase. In patients with TSH insufficiency the dose was adjusted to obtain stable free T4 concentrations in the upper half of the normal range. In patients with ACTH-insufficiency 10 mg hydrocortisone was administered p.o. 30 minutes before physical activity.

Patients with GHD were instructed in self-administration of GH using a pen device (Genotropin-Pen, Pfizer, Switzerland). Usual clinical care was provided with monthly visits including clinical assessment (side effects, weight, blood pressure, heart rate) and measurement of insulin-like growth factor 1 (IGF-1) concentrations in order to adjust GH doses. In male patients, GHRT was started with a dose of 0.1 mg sc/day. for two weeks followed be 0.2 mg sc./day for two weeks. In female patients the starting dose was 0.2 mg sc./day for two weeks followed by 0.4 mg/day for two weeks. In male and female patients the dose of GH was increased by 0.1 or 0.2 mg sc./day according to the monthly assessment of IGF-1 concentrations. The aim of this titration was an IGF-1 concentration in the upper half of the age-adjusted reference range after 3 months. After 6 months of GHRT, weight maintaining diet (total need of calories/d calculated according to the formula of Harris-Benedict, qualitatively consisting of 50% carbohydrate, 30% fat, and 20% protein), and identical physical activity as assessed by pedometer, the same studies were performed in the patients.

The patients attended the endocrine investigation unit for three visits before and three visits after 6 months GHRT, the control subjects were investigated once (three visits). The maximal time interval between visits was 7 days.

### Visit 1: Determination of VO_2max_ on a treadmill

Participants attended the endocrine investigation unit after having fasted for at least 4 hours. All volunteers had restrained from physical activity for 72 hours before the test. Body weight was measured on an electronic balance with subjects wearing light clothes and no shoes. Height was assessed by a stadiometer. BMI was calculated as the weight divided by the square of the height. End-expiratory waist circumference was measured with a flexible tape placed on a horizontal plane at the level of the iliac crest. Maximal aerobic exercise capacity was determined during an incremental workload test on a treadmill (CARDIOVIT AT-104 PC Ergo-Spirometrie, Schiller, Baar, Switzerland) until exhaustion. Increase of workload was chosen according to the estimated fitness status in order to obtain an exercise time of 9–12 minutes. During the test expired oxygen, carbon dioxide content and minute ventilation were measured continuously (Oxycon alpha, Jaeger, Würzburg, Germany). Furthermore, blood pressure was measured every two minutes and subjective level of exhaustion was assessed with the Borg scale. Maximal exercise aerobic capacity at exhaustion was documented, in parallel with the heart rate at this time point (=100%). Fifty% of maximal heart rate was calculated.

After a short break, the subjects were jogging for 30–60 minutes on the treadmill. This exercise aimed at determining the velocity and gradient of the treadmill at which the subject exhibited a heart rate that corresponded to 50% of heart rate at maximal oxygen consumption (VO_2max_). Furthermore, participants were able to get familiar with the protocol of visit 3.

### Visit 2: Two-step euglycaemic hyperinsulinaemic clamp

Hepatic and peripheral insulin sensitivity was determined with a two-step hyperinsulinaemic-euglycaemic clamp^14^. Participants attended the investigation unit at 07:30 am after an overnight fast. They had restrained from physical activity for the previous 72 hours and avoided alcohol consumption for 48 hours before the test. On arrival, subjects were asked to void. After that, they rested quietly in a bed in a semisupine position. A cannula was retrogradely inserted into the vein of the left wrist for blood sampling. A second cannula was inserted into an antecubital vein of the other arm for glucose, insulin, and tracer infusions. Blood was collected at baseline for biochemical evaluation. Whole-body glucose turnover was assessed in the basal condition and after a 2-h 6,6-[2H2]glucose infusion (bolus: 3 mg/kg; continuous: 15 μg/kg/min). Afterwards, a 2-step hyperinsulinaemic euglycaemic clamp was performed (Insulin infusion: 0.2 and 1 mU/kg/min, 90 min each)^14^. Stable glycaemia of 5.0 mmol/L was achieved by variable infusion of 20% dextrose according to the actual plasma glucose value which was obtained every five minutes with a bedside glucose meter (YSI2300; Yellow Springs Instruments, Yellow Springs, OH, USA). The clamp was performed in combination with measures of hepatic glucose output during the last 30 minutes of each step of the clamp. The infusion of 6,6-[2H2]glucose was altered in order to maintain a stable enrichment during the clamp.

### Visit 3: Measurement of IMCL and IHCL before and after 2 hours physical exercise on a treadmill at 50% of VO_2max_. Determination of subcutaneous (SAT) and visceral adipose tissue (VAT)

During 1.5 days previous to visit 3, subjects followed a fat rich diet consisting of a supplementary fat intake of 0.75 g fat/kg/body weight, administered as 3 additional snacks per day until the first ^1^H-MR-spectroscopy measurements. This protocol had previously been shown to efficiently replete IMCL stores in a standardized way^7^,^15^. Upon arrival, they received an additional standardized light meal. Afterwards IMCL and IHCL were determined by ^1^H-MR-spectroscopy immediately before and after a two hours walk on the treadmill at a workload of 50% of their VO_2max_. IHCL was determined first, while IMCL were determined ~30 minutes later. The exercise-induced changes in IMCL and IHCL are defined as ∆IMCL and ∆IHCL, respectively. The same day, SAT and VAT was determined using MR-imaging as described previously^16^.

### Biochemical analysis

Blood glucose levels were measured by a glucose-oxidase method using YSI2300 (Yellow Springs Instruments, Yellow Springs, OH, USA). Insulin concentrations were measured with electro-chemiluminescence immunoassays (Roche Modular-E170; Roche Diagnostics, Rotkreuz, Switzerland). The intra-assay CV was 1.1%, the inter-assay CV was 3.6%

### Assessment of hepatic and whole body insulin sensitivity and tracer calculations

Isotopes were bought from Cambridge Isotope Laboratories, Innerberg, Switzerland. Sterile pathogen-free solutions were prepared by the University Hospital Pharmacy, CHUV, Lausanne, Switzerland. Isotopic enrichments of D-[6-6-2H2]glucose were measured by gas-chromatography mass-spectrometry (GC 5890/MS 5971; Hewlett-Packard, Palo Alto, CA, USA). At rest, glucose rate of appearance (Ra) and rate of disappearance (Rd) were calculated from D-[6-6-2H2] glucose enrichments using Steele’s equations^17^. Assuming steady state conditions (plasma glucose variation <5%, variation of dextrose 10% infusion <5%) the same equation was used during the last 30 minutes of the first clamp step in order to calculate endogenous glucose production, which was calculated from the infusion rate and the total Ra. As a measure of hepatic insulin sensitivity we show the percentage of suppression of endogenous glucose production (EGP) in the steady state of the first clamp step compared with the baseline value.

Whole body insulin sensitivity is expressed as glucose infusion per kg body weight during the last 30 minutes of the second clamp step (M-value).

### Measurement of IMCL and IHCL

All MR examinations were done on a 3 Tesla system (TIM TRIO, SIEMENS Erlangen). For the measurement of IMCL in m. tibialis anterior, an extremity coil and a PRESS sequence (TR 3s, TE 30ms, 11 × 12 × 18 mm^3^, 128 scans) were used, while for determination of IHCL in segment 6 of the liver a body receive array coil with a STEAM sequence (TR 5s, TE 20ms, 20 × 30 × 30 mm^3^, average of 10 separate non-water-suppressed acquisitions during flat breathing) was applied, as previously reported^4^. Repositioning of the volunteer and placement of the coil for the second measurement after the exercise were monitored on localizer images. For the skeletal muscle, the bottom of the kneecap (patella) and for the liver, the lower part of the throat (fossa jugularis) was used as a fixation point for the repositioning. Spectra were analyzed in jMRUI^18^. The 10 acquisitions for IHCL were separately fitted with a fixed pattern of 5 lipid resonances for the region 0.9 to 2.8 ppm. 10 separate acquisitions (1 × 8 averages, 8 × 1 average, and 1 × 32 averages) were used to detect and balance problems of low signal-to-noise (with 1 average) and motion-induced dephasing (with multiple averages). IHCL results are expressed in signal percent, calculated as % area of the lipid resonances divided by the total signal area (water and lipid resonances in the same, non-water suppressed spectrum). No corrections for relaxation effects were made. IMCL concentrations (in millimoles per kilogram wet weight) were evaluated based on the fully relaxed, unsuppressed water signal as internal concentration standard as previously described^19^.

### Subcutaneous (SAT) and visceral adipose tissue (VAT) assessment

MR-imaging was performed with the body coil as combined transmit/receive coil, as previously described^16^. Briefly, in order to determine VAT and SAT, images were taken in axial direction with a T1-weighted fast spin echo technique (TR = 452 ms, TE = 38 ms, echo train length = 7, slice thickness of 10 mm, five slices per sequence, spacing between slices 20 mm, FOV 50 cm, image resolution 2 mm per pixel) from fingers to toes leading to 100–130 axial images per subject. Image analysis for volume determination was done by using a home-built program (MATLAB R2007a, The MathWorks, Natick, MA, USA) which is based on an extended point counting method and three sequential steps for the determination of visceral and subcutaneous adipose tissue^16^: i) the region of visceral fat is separated from subcutaneous fat by a simple contour line, ii) the points for the point counting method are set or deleted by the program based on a threshold value, and iii) visual inspection of the points lets the operator correct for intensity variations resulting from radio frequency inhomogeneity.

### Statistical analysis

Statistical analysis was performed on SPSS 22.0 (SPSS, Inc., Chicago, IL, USA). Results are expressed as median and interquartile range. A test for normality of the distribution (Kolmogorov-Smirnov) showed non-normal distributions for multiple parameters in sub-groups GHD, GHRT, or CS. In addition, the number of enrolled patients and thus of acquired data sets was relatively small (10 or below) and, therefore, non-parametric tests were used consequently to test differences and correlations. Non-parametric paired tests (Wilcoxon Signed Rank Test) were applied to analyze pre- and post-exercise IMCL and IHCL in controls and patients pre- and post GHRT, respectively. Non-parametric paired testing (Wilcoxon Signed Rank Test) was also applied to analyze differences between age/gender/waist-matched GHD and CS. In patients with GHD and CS together univariate non-parametric regression analysis (Kendall’s Tau) was performed to examine the influence of fat mass (i.e. SAT and VAT), exercise parameters (VO_2max_) and insulin sensitivity (hepatic and peripheral) on ∆IHCL and ∆IMCL. The number of successfully acquired data sets are indicated in the respective tables. A p-value< 0.05 was considered to be significant.

## Results

### Clinical and biochemical findings (Tables 1, 2)

The clinical and biochemical characteristics of patients and controls are summarized in Tables 1, 2. GHD patients and CS did not differ with regard to the matching criteria gender, age, BMI and waist. IGF-1 concentrations were significantly lower in GHD patients compared to CS. GHRT resulted in a significant increase in IGF-1 in patients with GHD without significant side effects.

The final dose of GH was attained at 3 months. During the remaining 3 months, a stable GH dose was administered. The median dose was 0.5 mg/day in male (range 0.3–0.6 mg/day) and 0.7 mg/day (range 0.5–1.2 mg/day) in female patients.

All except one female patient suffered from LH/FSH insufficiency, two of them were postmenopausal. One of the postmenopausal patients was substituted with a combination including estradiol valeras and cyproteron acetas, one premenopausal female patient was treated with a anticonceptive combination therapy including gestodenum 0.075 mg and ethinylestradiol 0.02 mg. All the male patients (n = 6) were substituted with testosterone undecanoas 1000 mg every 10–12 weeks. T4 replacement therapy was necessary in half of the patients. The dose ranged between 0.075 and 0.175 (median 0.1 mg). Cortisol replacement therapy was initiated in half of the patients. The dose of cortisol was adapted to the body weight and administered in two or three daily doses. The usual daily cortisol replacement dose used in the GHD patients was between 15 and 20 mg (median 17.5 mg/day).

### Fat mass (SAT, VAT; Table 2)

Data on GHRT induced changes in body composition had been published previously^20^. Briefly, GHD patients had similar SAT and VAT compared with CS. GHRT resulted in a significant decrease in SAT (p = 0.008) and in VAT (p  =  0.05).

### Exercise parameters and food intake (Table 1)

Time or intensity of physical activity during daily life was not significantly different in patients before and after GHRT as assessed by the physical activity diaries and pedometer recordings. Similarly, food intake was standardized in patients before and after GHRT as well as in controls and was not significantly different with regard to qualitative and quantitative food intake as assessed by food diaries.

VO_2max_ tended to be lower in GHD compared with CS (p = 0.10). GHRT resulted in a significant increase in VO_2max_ in patients with GHD.

### Glucose metabolism and insulin sensitivity (Table 1)

The insulin induced suppression of endogenous glucose production (EGP) during low dose insulin infusion was more pronounced GHD patients, but not statistically significantly different from CS. Glucose and insulin concentrations were not different during the two-step hyperinsulinemic euglycemic clamp procedures in patients with GHD before and after GHRT and in CS. Peripheral glucose uptake as assessed by the M-values was not different between GHD patients and CS. GHRT did neither result in significant changes in the suppression of EGP nor in the M-values.

### IMCL and IHCL (Table 3, Fig. 1)

IMCL pre-exercise were not significantly different in GHD patients before and after GHRT and in CS. A 2 h acute aerobic exercise tended to decrease IMCL in GHD (p = 0.069) and significantly decreased IMCL in patients after GHRT and in CS. ΔIMCL were not statistically different between GHRT, GHD, or CS.

IHCL pre-exercise were not statistically different in GHD patients before and after GHRT and in CS. IHCL significantly increased in all three groups following exercise. ΔIHCL were not statistically different between GHRT, GHD, or CS.

### Univariate non-parametric correlation between changes in IMCL (ΔIMCL) and IHCL (ΔIHCL), and parameters of exercise capacity, fat availability and insulin sensitivity in patients with GHD and in CS (Table 4)

ΔIMCL was not significantly correlated with exercise capacity, fat mass (SAT or VAT) or insulin sensitivity. ΔIHCL was positively correlated with fat mass that reached significance for VAT. In turn, ΔIHCL was negatively correlated with insulin sensitivity, yet reaching significance only for peripheral insulin sensitivity. Exercise capacity did not significantly correlate with ΔIHCL.

## Discussion

The main findings of this study can be summarized as follows: 1) A 2h-acute physical aerobic exercise at 50% of VO_2max_ results in a decrease in IMCL and a significant increase in IHCL in GHD patients (before and after GHRT) and in CS over a range of peak aerobic capacity, fat mass and insulin sensitivity. These findings indicate that flexibility of ectopic lipids is not only restricted to healthy physically active subjects but is also a phenomenon which can be documented in hypopituitary patients with GHD before and after GHRT 2) In GHD and CS the exercise-induced increase in IHCL is significantly related to fat mass, in particular VAT, and insulin sensitivity but we did not find any relation with peak aerobic capacity. The exercise-induced changes in IMCL are not significantly related to insulin sensitivity, peak aerobic capacity or fat mass. 3) GHRT significantly increases exercise capacity without significantly affecting the flexibility of ectopic lipids or insulin sensitivity.

IMCL depletion following physical exercise occurred in GHD patients before and after GHRT as well as in CS, corroborating the importance of skeletal muscle activity in regulating IMCL^21^,^22^. Quantitatively, the current IMCL repletion and depletion following standardized diet and physical exercise, respectively, is consistent with previous findings from our group^7^,^15^ and others^23^,^24^. Interestingly, ΔIMCL was not significantly correlated with peak aerobic capacity, fat mass or insulin sensitivity indicating that other regulatory factors may play a role. Since skeletal muscle is known to exhibit a significant plasticity by adapting to endurance exercise training with an increase in mitochondrial volume, to strength training with an increase in myofibrillar volume and to high fat diet with an increase in IMCL^25^, it is conceivable that other factors are more important in regulating ΔIMCL than exercise capacity, fat mass and insulin sensitivity. Despite the fact that GH is an important lipolytic hormone and its target for the degradation of IMCL, the hormone sensitive lipase, has been shown to be expressed in skeletal muscle^15^, no significant effect of GHRT on the flexibility of IMCL could be documented. This finding is intriguing but support the fact that IMCL and their flexibility is not only regulated by a systemic hormonal effect.

In contrast to IMCL, IHCL significantly increased in patients (before and after GHRT) as well as in CS after an acute bout of 2 h aerobic physical exercise suggesting that IHCL, like IMCL, are flexible fuel stores. This is consistent with recent data in physically active healthy subjects^3^,^5^,^6^ indicating that flexibility of IHCL is not only restricted to healthy physically active subjects but is also a phenomenon that can be documented in hypopituitary patients with GHD before and after GHRT. It is likely that IHCL are flexible stores that increase in conditions of increased release of FFA from adipose tissue as during a 2 h exercise bout^3^. An increase in FFA release can not only be documented during physical exercise, but also during starvation. Indeed, IHCL has been shown to increase during short-term starvation with a concomitant increase in FFA^26^ further supporting our results. Consistent with these findings it has been shown that the hepatocellular uptake of FFA is selectively preserved during starvation^27^. In addition, a mixed diet, consisting of 50% carbohydrate, 35% fat and 15% protein and an iso-caloric fat rich (83%) low carbohydrate (2%) and protein (15%) diet resulted in a similar exercise-induced increase in IHCL in six healthy trained male volunteers, further corroborating our results^6^.

Interestingly, ΔIHCL is not significantly related to aerobic peak capacity but positively correlated with fat mass, in particular VAT in patients with GHD and in CS. This is a new finding and we can only speculate about the underlying mechanisms. It may suggest that physical exercise induces lipolysis as evidenced by an increase in systemic FFA^3^. This increase is positively related to the available fat mass and not to aerobic peak exercise capacity. The exercise-induced increase in lipolysis outweighs the cellular transport capacity of FFA into the peripheral tissues resulting in an increase in plasma FFA concentrations^22^. A part of this excess of FFA is taken up by the liver. The liver, in turn, may be considered as a sink that can stock the excess of FFA as IHCL^28^. So far, the variability of IHCL between different subjects has been shown to be explained in part by the amount of VAT^29^,^30^,^31^, in keeping with the present exercise-induced changes in IHCL. A possible explanation may be that the exercise-induced lipolysis results in a FFA flux directly from the VAT through the portal system to the liver thereby explaining the significant relationship between VAT and ΔIHCL. In contrast, FFAs originating from SAT, have to cross a capillary bed before potentially arriving to the liver. The relationship between SAT and ΔIHCL might, therefore, be less straightforward. Interestingly, a similar exercise challenge - as performed in our study - but with a parallel administration of glucose (thereby eliciting an insulin response) blunted the exercise-induced increase in FFA and in parallel the increase in IHCL suggesting that, indeed, the availability of FFAs may be critical for the observed increase in IHCL^5^. However, FFA-turnover studies would be needed to confirm this hypothesis. In addition, ΔIHCL was negatively correlated with insulin sensitivity, in particular with peripheral insulin sensitivity. This finding is not surprising if we assume that fat mass and FFA have a significant impact on insulin action at the skeletal muscle^32^,^33^,^34^.

GHD tend to have a reduced peak aerobic capacity and GHRT resulted in an increase in peak aerobic capacity, in keeping with previous results^9^,^35^. However, the flexibility of ectopic lipids was not significantly influenced by GHRT. This finding indicates that the potential GH-induced increased lipolytic action, especially at the skeletal muscle in order to reduce IMCL, is only of minor importance and other effects of GHRT like the well-known increase in lean body mass^9^, the increase in oxygen transport capacity as assessed by an increase in red cell mass^2^ and the improvement of endothelial function^36^,^37^, thereby reducing peripheral vascular resistance, are probably more important parameters to improve exercise performance than a potential metabolic effect on ectopic lipids.

In contrast to previous studies^9^ the current data indicate that GHD patients are not insulin resistant when compared to age, gender, BMI and waist matched control subjects with comparable VAT. The first line of evidence is provided by the clamp data that were similar between GHD and CS. The second line of evidence concerns the ectopic lipid levels which were alike between patients and CS (Table 2). If we assume that ectopic lipids are related to insulin sensitivity, these findings are in keeping with the clamp data. The additional matching criteria “waist circumference”, usually not included in previous studies^9^, may contribute to these results. Finally, our data are in line with recent data that investigated ectopic lipids in addition to SAT and VAT in GHD before and after GHRT as well as in CS. These results suggest that GHD patients tend to have even lower ectopic lipids than CS^12^.

This study has its limitations: 1) We were only able to include totally 10 patients and 10 CS. This was essentially related to the time consuming investigations and the strict matching criteria between GHD patients and control subjects. It may, therefore, be that more subtle differences between the groups were not detected or just failed the significant statistical level. However, we believe that the important differences between the different groups could be identified as shown in previous studies with a similar number of volunteers^23^,^24^. 2) We cannot exclude that without matching for waist and, therefore, for VAT, the results may have been different. An extended study would be necessary to address this interesting question 3) We are aware that short-time fat rich diet can result in an increase in IHCL. However, quantitatively the supplied additional snacks were in the order of magnitude of previous studies^6^,^38^ where no significant effect on IHCL could be demonstrated. Furthermore, the main aim of the current study was to determine whether GHRT has an effect of ectopic lipids. In in this context standardization of dietary regimen is of primordial importance^39^. 4) We are aware that correlations are never a proof of an underlying mechanism and the results have to be interpreted carefully. 5) It is known that GH-induced water retention could be a confounder in an impedance based assessment of body composition in GHD patients, including LBM. However, since the focus of this study is mainly on fat mass (SAT and VAT) using MRI method and not on LBM, we do not believe that the conclusions are strongly affected by the influence of LBM.

In conclusion, this study suggests that in patients with GHD (before and after GHRT) IMCL and IHCL are flexible ectopic lipid stores which are acutely regulated by physical exercise, albeit in different directions. Fat mass and insulin sensitivity appear to influence the increase in IHCL after exercise whereas the changes of IMCL are independent of fat mass, insulin sensitivity and exercise capacity. GHRT does not influence ectopic lipids or their flexibility but improves exercise capacity.

## Additional Information

**How to cite this article**: Christ, E. R. *et al.* Effects of aerobic exercise on ectopic lipids in patients with growth hormone deficiency before and after growth hormone replacement therapy. *Sci. Rep.* **6**, 19310; doi: 10.1038/srep19310 (2016).

## Figures and Tables

**Figure 1:**
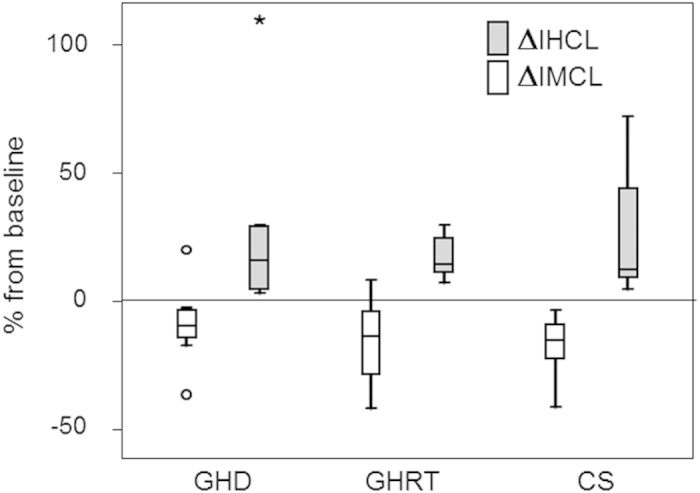
Exercise induced changes in IMCL and IHLC in control subjects and in patients with GHD before and after GHRT. This figure depicts the exercise-induced changes in ectopic lipids (ΔIMCL and ΔIHCL) in sedentary control subjects (CS), patients before (GHD) and after (GHRT) growth hormone replacement therapy. The values are given in % from baseline and the number of values used for the individual bars is listed in Table 3. Consistently, a 2 h- aerobic exercise resulted in a decrease in IMCL and an increase in IHCL in CS, patients before (GHD) and after GHRT. ΔIMCL and ΔIHCL were not significantly different between CS and GHD. GHRT did not significantly influence ΔIMCL and ΔIHCL.

**Table 1:** Diagnostic and therapeutic characteristics of patients with GHD

Patient No.	Gender	Diagnosis	Treatment			Additional Hormonedeficiencies		
			Surg	DxRT	DA	ACTH	TSH	LH/FSH
1	M	hormone-inactive pituitary adenoma	+	+		+	+	+
2	M	hormone-inactive pituitary adenoma	+	+		+	+	+
3	F	epidermoid cyst	+			+		+
4	M	gonadotroph cell adenoma	+					+
5	F	idiopathic GH deficiency						+
6	M	hormone-inactive pituitary adenoma	+			+	+	+
7	M	prolactinoma			+	+	+	+
8	F	hormone-inactive pituitary adenoma	+				+	+
9	M	macroprolactinoma			+			+
10	F	hormone-inactive pituitary adenoma	+					

**Table 2:** Clinical and biochemical characteristics, fat mass, exercise capacity, and insulin sensitivity in patients with GHD before and after GHRT and in control subjects

Clinical Parameters	GHD (pre-treatment)	GHRT (post-treatment)	CS	p *GHD vs.GHRT*	p *GHD vs.CS*
Gender	4F	4F	4F	ns	ns
	6M	6M	6M	(n = 10/10)	(n = 10/10)
Age (years)	45.5 (n = 10)	46.1 (n = 10)	44.3 (n = 10)	p = 0.005	ns
	(32.0, 54.6)	(32.6, 55.1)	(30.6, 53.6)	(n = 10/10)	(n = 10/10)
Weight (kg)	76.3 (n = 10)	76.5 (n = 10)	70.5 (n = 10)	ns	ns
	(65.0, 90.7)	(64.3, 88.3)	(58.5, 95.7)	(n = 10/10)	(n = 10/10)
LBM (kg)	50.1 (n = 10)	48.3 (n = 9)	49.8 (n = 10)	0.015	ns
	(42.7, 61.5)	(43.0, 60.9)	(38.5, 63.8)	(n = 9/9)	(n=10/10)
BMI (kg/m^2^)	27.3 (n = 10)	27.3 (n = 10)	24.0 (n = 10)	ns	ns
	(22.9, 29.0)	(22.3, 29.8)	(22.0, 29.2)	(n = 10/10)	(n = 10/10)
Waist (cm)	91.6 (n = 10)	91.3 (n = 10)	90.0 (n = 10)	ns	ns
	(82.3, 100.3)	(81.3, 95.3)	(80.5, 97.9)	(n = 10/10)	(n = 10/10)
IGF-1 (nmol/L)	68.5 (n = 10)	153.0 (n = 10)	111.5 (n = 10)	p = 0.005	p = 0.001
	(40.3, 87.6)	(112.0, 174.3)	(93.8, 138.3)	(n = 10/10)	(n = 10/10)
**Body composition**					
SAT (kg)	16.5 (n = 10)	14.7 (n = 9)	15.2 (n = 10)	p = 0.008	ns
	(14.0, 22.2)	(10.8, 21.7)	(12.2, 23.4)	(n = 9/9)	(n = 10/10)
VAT (kg)	2.37 (n = 10)	1.96 (n = 9)	2.57 (n = 10)	p = 0.05	ns
	(1.49, 3.34)	(1.09, 2.62)	(1.31, 3.37)	(n = 9/9)	(n = 10/10)
**Exercise capacity**					
VO_2max_ (ml/kg/min)	36.9 (n = 10)	42.1 (n = 10)	39.9 (n = 10)	p = 0.005	ns
	(29.0, 40.1)	(37.6, 48.5)	(36.2, 46.5)	(n = 10/10)	(n = 10/10)
**Insulin sensitivity**					
Suppression EGP from baseline (%: low insulin dose)	59.1 (n = 9)	40.5 (n = 8)	38.5 (n = 10)	ns	ns
	(25.6, 69.5)	(31.6, 52.9)	(16.7, 70.0)	(n = 8/8)	(n = 9/9)
M-value (high insulin-dose) (mg/kg/min)	7.36 (n = 9)	7.50 (n = 10)	7.06 (n = 10)	ns	ns
	(6.84, 8.62)	(7.08, 7.99)	(5.28, 9.50)	(n = 9/9)	(n = 9/9)

Values are median and interquartile range.

GHD = growth hormone deficiency; GHRT = parameter after growth hormone replacement therapy; CS = control subjects; SAT = subcutaneous adipose tissue; VAT = visceral adipose tissue; LBM = lean body mass. Suppression EGP = suppression of endogenous glucose production = measure of hepatic insulin resistance; M-value = glucose infusion at high insulin dose = measure of peripheral insulin resistance.

Differences were evaluated by a non-parametric paired test (Wilcoxon Signed Ranks Test), between GHD patients (GHD) before and after therapy for individual patients and between GHD and CS for age/gender/waist-matched pairs, respectively.

**Table 3:** Ectopic lipids before and after exercise in patients with GHD before and after GHRT and in control subjects

MRS	GHD (pre-treatment)		GHRT (post-treatment)		CS	
		p (pre-exercise vs. post)		p (pre-exercise vs. post)		p (pre-exercise vs. post)
IMCL pre-exercise(mmol/kg wet weight)	2.9 (n = 8)(2.6, 4.5)		3.9 (n = 8)(2.5, 5.7)		3.6 (n = 9)(2.7, 6.2)	
IMCL post-exercise(mmol/kg wet weight)	2.5 (n = 8)(2.4, 3.8)	p = 0.069 (n=8/8)	3.4 (n = 8)(1.6, 4.9)	p = 0.036 (n = 8/8)	3.2 (n = 9)(2.3, 5.2)	p = 0.008 (n = 9/9)
∆ IMCL(% from baseline)	−9.4 (n = 8)(−15.7, −2.9)		−13.5 (n = 8)(−35.0, −3.5)		−15.4 (n = 9)(−27.9, −8.2)	
IHCL pre-exercise(% from total signal)	2.1 (n = 10)(1.0, 6.9)		1.9 (n = 9)(1.2, 3.8)		4.0 (n = 10)(1.5, 23.5)	
IHCL post-exercise(% from total signal)	2.4 (n = 10)(1.7, 8.0)	p = 0.005 (n = 10/10)	2.1 (n = 9)(1.4, 4.4)	p = 0.008 n = (9/9)	4.3 (n = 10)(2.1, 25.2)	p = 0.009(n = 10/10)
∆ IHCL(% from baseline)	23.2 (n = 10)(5.2, 35.6)		15.9 (n = 9)(11.4, 28.7)		11.8 (n = 10)(5.6, 47.1)	

Values are median and interquartile range. All comparisons between groups GHD, GHRT, and CS were non-significant.

GHD = patient with growth hormone deficiency; GHRT = patients after growth hormone replacement therapy; CS = control subjects; MRS = magnetic resonance spectroscopy; IMCL = intramyocellular lipids; IHCL = intrahepatocellular lipids; ∆IMCL and ∆IHCL = difference of intramyocellular and intrahepatocellular lipids before and after 2 h exercise at 50–60% VO_2max_.

Differences between groups and between pre and post were evaluated by a non-parametric paired test (Wilcoxon Ranked Sign Test).

**Table 4:** Univariate non-parametric regression of ∆IMCL and ∆IHCL with fat mass (subcutaneous adipose tissue and visceral adipose tissue), insulin sensitivity and exercise capacity (VO_2max_) in patients with GHD and CS subjects.

	Correlationcoefficient	p-values	Correlationcoefficient	p-values
	vs. ∆IMCL		vs. ∆IHCL	
Fat mass				
Subcutaneous adipose tissue	0.02	0.93(n = 17)	0.18	0.27(n = 20)
Visceral adipose tissue	0.15	0.41(n = 17)	0.44	**0.005**(n = 20)
Insulin sensitivity				
Supp. EGP(low insulin dose)	0.20	0.28(n = 16)	−0.24	0.15(n = 19)
M-value(high insulin dose)	0.15	0.42(n = 16)	−0.37	**0.03**(n = 19)
Exercise parameters				
VO_2max_	0.03	0.87(n = 17)	0.20	0.22(n = 20)

Correlation analysis (Kendall’s tau) was performed with data of patients with GHD and CS together.

Suppression EGP = suppression of endogenous glucose production = measure of hepatic insulin resistance; M-value = glucose infusion at high insulin dose = measure of peripheral insulin resistance; ∆IMCL/∆IHCL = 2 h-aerobic exercise induced changes of IMCL/IHCL.

The shaded boxes show positive correlations between fat availability and ΔIHCL, in particular for visceral fat; and negative correlations between insulin sensitivity and ΔIHCL. In contrast, the correlations with ΔIMCL are either very weak or positive.
